# Repeated vital sign measurements in the emergency department predict patient deterioration within 72 hours: a prospective observational study

**DOI:** 10.1186/s13049-018-0525-y

**Published:** 2018-07-13

**Authors:** Vincent M. Quinten, Matijs van Meurs, Tycho J. Olgers, Judith M. Vonk, Jack J. M. Ligtenberg, Jan C. ter Maaten

**Affiliations:** 1 Department of Emergency Medicine, University of Groningen, University Medical Center Groningen, HPC TA10, PO Box 30001, 9700 RB Groningen, The Netherlands; 2 Department of Critical Care, University of Groningen, University Medical Center Groningen, Groningen, The Netherlands; 3 Department of Pathology and Medical Biology, Medical Biology section, University of Groningen, University Medical Center Groningen, Groningen, The Netherlands; 4 Department of Epidemiology, University of Groningen, University Medical Center Groningen, Groningen, The Netherlands

**Keywords:** Sepsis, Accident & emergency medicine, Patient deterioration, Vital signs

## Abstract

**Background:**

More than one in five patients presenting to the emergency department (ED) with (suspected) infection or sepsis deteriorate within 72 h from admission. Surprisingly little is known about vital signs in relation to deterioration, especially in the ED. The aim of our study was to determine whether repeated vital sign measurements in the ED can differentiate between patients who will deteriorate within 72 h and patients who will not deteriorate.

**Methods:**

We performed a prospective observational study in patients presenting with (suspected) infection or sepsis to the ED of our tertiary care teaching hospital. Vital signs (heart rate, mean arterial pressure (MAP), respiratory rate and body temperature) were measured in 30-min intervals during the first 3 h in the ED. Primary outcome was patient deterioration within 72 h from admission, defined as the development of acute kidney injury, liver failure, respiratory failure, intensive care unit admission or in-hospital mortality. We performed a logistic regression analysis using a base model including age, gender and comorbidities. Thereafter, we performed separate logistic regression analyses for each vital sign using the value at admission, the change over time and its variability. For each analysis, the odds ratios (OR) and area under the receiver operator curve (AUC) were calculated.

**Results:**

In total 106 (29.5%) of the 359 patients deteriorated within 72 h from admission. Within this timeframe, 18.3% of the patients with infection and 32.9% of the patients with sepsis at ED presentation deteriorated. Associated with deterioration were: age (OR: 1.02), history of diabetes (OR: 1.90), heart rate (OR: 1.01), MAP (OR: 0.96) and respiratory rate (OR: 1.05) at admission, changes over time of MAP (OR: 1.04) and respiratory rate (OR: 1.44) as well as the variability of the MAP (OR: 1.06). Repeated measurements of heart rate and body temperature were not associated with deterioration.

**Conclusions:**

Repeated vital sign measurements in the ED are better at identifying patients at risk for deterioration within 72 h from admission than single vital sign measurements at ED admission.

## Background

More than one in five patients presenting to the emergency department (ED) with (suspected) infection or sepsis deteriorate within 72 h from admission, despite treatment [1]. Recent advances in research have improved our understanding of the pathophysiology of sepsis [2]. The adoption of surviving sepsis campaign (SSC) guidelines, increased awareness and early goal-directed therapy dramatically reduced sepsis-related mortality over the past two decades [3, 4]. However, one of the main challenges for the physician in the ED remains to determine the risk of deterioration for the individual patient [2]. The numerous sepsis-related biomarkers lack sensitivity and specificity for deterioration and are not readily available in the ED [5–7]. Despite the relative ease of measurement, surprisingly little is known about vital signs in relation to clinical outcomes, especially in the ED setting [8–11]. There is limited evidence that oxygen saturation and consciousness level at ED arrival are associated with mortality, and that heart rate and Glasgow coma scale (GCS) are associated with intensive care unit (ICU) admission [9, 11]. For all other vital signs, insufficient evidence is available [9, 11]. The few available studies mostly studied vital signs used in triage systems or vital signs obtained at the time of ED admission [9, 12]. Almost one third of the medical patients who arrive at the ED with normal vital signs show signs of deterioration in vital signs within 24 h [13]. Our pilot study in the ED showed that vital signs change significantly during the patient’s stay in the ED [7]. However, surprisingly little is known on how to monitor and identify deteriorating patients in the emergency department [13]. The latest SSC guidelines recommend a thorough re-evaluation of routinely measured vital signs as parameter for response to treatment [4]. Therefore, the aim of the current study was to determine whether repeated vital sign measurements during the patient’s stay in the ED can distinguish between patients who will deteriorate within 72 h from admission and patients who will not.

## Methods

### Study design and setting

This study is a predefined prospective observational study, part of the Sepsis Clinical Pathway Database (SCPD) project in our emergency department (ED). The SCPD project is a prospective cohort study of medical patients presenting to the ED with fever and/or suspected infection or sepsis. Data was collected in the ED of the University Medical Center Groningen in The Netherlands, an academic tertiary care teaching hospital with over 30,000 ED visits annually.

This study was carried out in accordance with the Declaration of Helsinki, the Dutch Agreement on Medical Treatment Act and the Dutch Personal Data Protection Act. The Institutional Review Board of the University Medical Center Groningen ruled that the Dutch Medical Research Involving Human Subjects Act is not applicable for this study and granted a waiver (METc 2015/164). All participants provided written informed consent.

### Study population

Data was collected between March 2016 and February 2017. Consecutive medical patients visiting the ED between 8 a.m. and 23 p.m. were screened for eligibility. Inclusion criteria were: (1) age of 18 years or older, (2) fever (> = 38 °C) or suspected infection or sepsis, (3) able to provide written informed consent. The clinical suspicion of infection or sepsis was judged by the coordinating internist acute medicine on duty. He/she handles all medical patient announcements from general practitioners or the emergency medical services (EMS), and medical patients that enter the ED without previous announcement. The judgement was based on information provided over the phone during the announcement, information obtained at triage and immediately after ED admission of the patient. Only patients with at least three repeated vital sign measurements during their first 3 h in the ED were included in the final analysis.

### Data collection

The data collected in the SCPD project includes socio-demographic information, patient history, prescription drug usage, comorbidity, treatment parameters, results from routine blood analysis, questionnaires about activities of daily living, follow-up during the patient’s stay in the hospital and registration of various endpoints. The data was collected by trained members of our research staff during the patient’s stay in the ED and combined with data from the patient’s medical record for follow-up during the patient’s stay in the hospital.

For the current study, next to the data collected for all patients included in the SCPD project, we repeatedly measured vital signs in 30-min intervals during the patient’s stay in de ED. These vital signs included heart rate, respiratory rate and blood pressure, measured using a Philips MP30 or MX550 bed-side patient monitor (Philips IntelliVue System with Multi-Measurement Module; Philips, Eindhoven, The Netherlands). Furthermore, the body temperature was measured using an electronic tympanic ear thermometer (Genius 2; Mountainside Medical Equipment, Marcy, New York, USA).

All patients received treatment for infection or sepsis as per our hospital’s standardized protocol at the treating physician’s discretion. This protocol included intravenous antibiotics, fluid resuscitation and oxygen supplementation [7]. The protocol did not change during the inclusion period and was not influenced by the patient’s participation in the study. For patients arriving at the ED with EMS and (suspected) sepsis, treatment with fluid resuscitation and supplementary oxygen was started in the ambulance by EMS personnel according to the nationwide EMS guidelines for sepsis in The Netherlands [14]. The average time from EMS dispatch call to ED arrival is 40 min in The Netherlands, but actual dispatch times in this study were not measured [14]. Pre-hospital start of treatment was not influenced by the patient’s participation in the study.

### Endpoints and definitions

The primary endpoint was patient deterioration within 72 h from ED admission. We defined patient deterioration as the development of organ dysfunction, ICU admission or death during the patient’s stay in the hospital. For organ dysfunction, we distinguished between acute kidney failure (AKI), liver failure and respiratory failure. AKI was defined using the Kidney Disease Improving Global Outcomes (KDIGO) criteria as an increase in serum creatinine by 26.5 μmol/L (0.3 mg/dL) within 48 h or 1.5 times the baseline (known or presumed to have occurred within the prior 7 days) [15]. Liver failure was defined as total bilirubin level > 34.2 μmol/L (2.0 mg/dL) and either alkaline phosphatase or a transaminase level above twice the normal limit [16]. Respiratory failure was defined as the need for mechanical ventilation, or either hypoxemia (PaO_2_ < 8.0 kPa) or hypercapnia (PaCO_2_ > 6.5 kPa) in the arterial blood gas analysis, or a peripheral oxygen saturation < 90% when breathing ambient air or < 95% with at least 2 L/min of oxygen supplementation [17]. In-hospital mortality was defined as all-cause mortality during the patient’s stay in the hospital. The Sepsis-2 criteria (2001 international sepsis definitions conference) were used to define sepsis, severe sepsis or septic shock, i.e. two or more systemic inflammatory response syndrome criteria and suspected/confirmed infection [18].

### Statistical analysis

Continuous data were reported as median with interquartile range (IQR) and analysed using the Mann-Whitney U test. Categorical data were summarized as counts with percentages and analysed using the Chi-square test.

For each vital sign and for each patient, we used the repeated measurements to estimate the linear change and variability over time. Linear change over time was estimated using individual linear regression analysis separately for each vital sign (heart rate, respiratory rate, mean arterial pressure and temperature) with the time of the measurement (in minutes) as independent variable. The resulting regression estimates for time, indicate the linear change per minute for each patient and each vital sign. The variability of each vital sign was calculated as the difference between the highest and lowest value during the first 3 h in the ED.

To analyse the added value of the linear change and variability over time of each vital sign as predictors for patient deterioration within 72 h, we performed multiple logistic regression analysis. First, we constructed a base model containing age, gender and comorbidity. The added value of each vital sign to the base model was assessed using the following logistic regression analyses: (1) base model + vital sign value at admission, (2) base model + vital sign value at admission + change of the vital sign during the first 3 h in the ED and (3) base model + vital sign value at admission + variability of the vital sign during the first 3 h in the ED. For each model, the area under the receiver operator curve (AUC) was calculated using the predicted probabilities.

All statistical analyses were performed using IBM SPSS Statistics for Windows V.23.0 (IBM Corp, Armonk, New York, USA). A two-tailed *p*-value of < 0.05 was considered significant.

## Results

### Patient characteristics

**Fig. 1:**
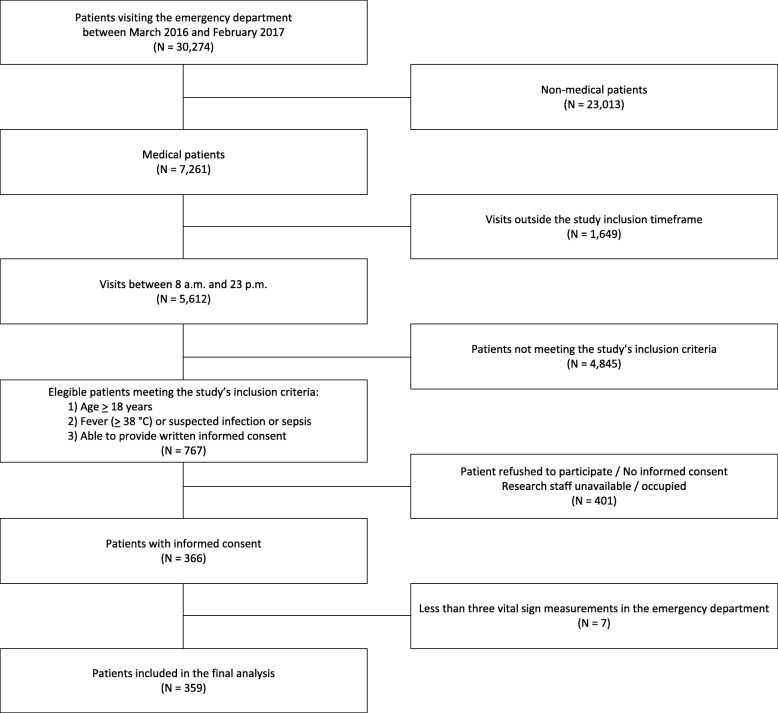
Flow chart of patient recruitment. Consecutive adult medical patients visiting the emergency department of the University Medical Center Groningen between March 2016 and February 2017 were screened for eligibility

During the study period 366 patients met the inclusion criteria (Fig. 1). Seven patients were excluded because they had less than three repeated vital sign measurements in the emergency department (ED) during the first 3 h from admission. The remaining 359 patients were included in the final analysis. Of the 359 patients, 106 (29,5%) patients deteriorated within 72 h from admission (Table 1). Patients with cardiac disease (*p* = 0.004), COPD (*p* = 0.047) or diabetes (*p* = 0.002), deteriorated more often compared to patients without these comorbidities. Malignancy (28.4%) and organ transplant (26.7%) were the most frequent comorbidities (Table 2).

**Table 1:** Patient characteristics

	Overall	Not deteriorated	Deteriorated	*p* Value
Number of patients [n (%)]	359 (100)	253 (70.5)	106 (29.5)	–
Demographics				
Age [median (IQR)]	63 (49; 71)	60 (47; 70)	66 (56; 74)	.001*
Male [n (% of the group)]	222 (61.8)	149 (58.9)	73 (68.9)	.076
Comorbidity				
Number of comorbidities [median (IQR)]	1 (0; 2)	1 (0; 2)	1 (1; 2)	.001*
Cardiac disease [n (% of the group)]	66 (18.4)	37 (14.6)	29 (27.4)	.004*
COPD [n (% of the group)]	23 (6.4)	12 (4.7)	11 (10.4)	.047*
Diabetes [n (%of the group)]	63 (17.5)	34 (13.4)	29 (27.4)	.002*
Chronic kidney disease [n (% of the group)]	43 (12.0)	26 (10.3)	17 (16.0)	.125
Chronic liver disease [n (% of the group)]	30 (8.4)	19 (7.5)	11 (10.4)	.370
Organ transplant [n (% of the group)]	96 (26.7)	64 (25.3)	32 (30.2)	.339
Malignancy [n (% of the group)]	102 (28.4)	77 (30.4)	25 (23.6)	.189
None of the above [n (% of the group)]	98 (27.3)	81 (32.0)	17 (16.0)	.002*
Disease severity				
Infection [n (% of overall)]	82 (22.8)	67 (81.7)	15 (18.3)	.011*
Sepsis [n (% of overall)]	277 (77.2)	186 (67.1)	91 (32.9)	.011*
Vital signs at ED admission				
Heart rate (bpm) [median (IQR)]	95 (83.0; 110.0)	95.0 (82.0; 110.0)	95.5 (83.0; 110.0)	.262
Mean arterial pressure (mmHg) [median (IQR)]	91.7 (83.3; 102.9)	94.3 (86.3; 103.3)	85.8 (73.4; 97.3)	<.001*
Respiratory rate (/min) [median (IQR)]	19.0 (16.0; 24.0)	18.0 (16.0; 23.3)	20.0 (17.0; 25.0)	.031*
Body temperature (°C) [median (IQR)]	37.8 (37.0; 38.6)	37.8 (37.0; 38.6)	38.0 (37.0; 38.8)	.564
Vital sign change				
Heart rate (bpm) [median (IQR)]	−1.10 (−2.89; 0.00)	−1.14 (− 2.89; 0.00)	−0.90 (− 2.93; − 0.01)	.810
Mean arterial pressure (mmHg) [median (IQR)]	− 0.97 (− 2.86; 0.57)	−0.91 (− 2.71; 0.50)	−1.18 (− 3.04; 0.84)	.833
Respiratory rate (/min) [median (IQR)]	− 0.07 (− 0.61; 0.58)	−0.09 (− 0.69; 0.61)	−0.07 (− 0.45; 0.56)	.427
Body temperature (°C) [median (IQR)]	−0.05 (− 0.17; 0.06)	−0.05 (− 0.17; 0.06)	−0.04 (− 0.17; 0.07)	.997
Vital sign variability				
Heart rate (bpm) [median (IQR)]	12.0 (7.0; 20.0)	12.0 (7.0; 19.5)	12.0 (7.0; 20.5)	.740
Mean arterial pressure (mmHg) [median (IQR)]	15.3 (9.7; 21.7)	14.0 (8.9; 19.5)	18.2 (12.6; 27.5)	<.001*
Respiratory rate (/min) [median (IQR)]	5.0 (2.0; 8.0)	4.0 (2.0; 7.0)	6.0 (3.0; 9.8)	.001*
Body temperature (°C) [median (IQR)]	0.7 (0.2; 1.1)	0.7 (0.4; 1.1)	0.7 (0.4; 1.2)	.512
Hospital admission				
Length of stay (days) [median (IQR)]	4.7 (0.7; 7.9)	3.6 (0.2; 6.2)	6.7 (4.1; 11.3)	<.001*
Mortality				
28-day [n (% of the group)]	17 (4.7)	4 (1.6)	13 (12.3)	<.001*
6-month [n (% of the group)]	44 (12.3)	24 (9.5)	20 (18.9)	.013*

COPD: chronic obstructive pulmonary disease; ED: emergency department; IQR: interquartile range

**Table 2:** Study population comorbidity matrix

*N* = 359	Cardiac disease	COPD	Diabetes	Chronic Kidney Disease	Chronic Liver Disease	Organ Transplant	Malignancy
Cardiac disease	66	10	15	13	3	16	17
COPD		23	3	1	1	4	5
Diabetes			63	7	9	17	11
Chronic Kidney Disease				43	2	29	4
Chronic Liver Disease					30	11	3
Organ Transplant						96	21
Malignancy							102

### Patient deterioration

Signs of organ failure were observed in 21.2% of the patients at ED admission (Table 3). An additional 6.1% of the patients deteriorated in the first 24 h after admission. The increase in respiratory failure (+ 4.2%) was the largest contributor to this deterioration. In the first 48 h after admission, 3.1% of the patients deteriorated to multiple organ failure. Most deterioration took place within the first 72 h from admission (+ 8.3%), with only a small increase (+ 1.7%) during the rest of the hospitalization.

**Table 3:** Patient deterioration outcomes in different timeframes during the patient’s stay in-hospital and divided by infection and sepsis on emergency department presentation

	Acute Kidney Injury	Liver failure	Respiratory failure	Organ failure		ICU admission	In-hospital mortality	Deteriorated
				Single	Multiple			
Total (N = 359, 100.0%)								
At ED admission	45 (12.5%)	21 (5.8%)	14 (3.9%)	72 (20.1%)	4 (1.1%)	–	–	76 (21.2%)
24 h after ED admission	51 (14.2%)	22 (6.1%)	29 (8.1%)	82 (22.8%)	10 (2.8%)	16 (4.5%)	1 (0.3%)	98 (27.3%)
48 h after ED admission	57 (15.9%)	23 (6.4%)	33 (9.2%)	83 (23.1%)	15 (4.2%)	18 (5.0%)	1 (0.3%)	102 (28.4%)
72 h after ED admission	60 (16.7%)	23 (6.4%)	35 (9.7%)	87 (24.2%)	15 (4.2%) ^x^	18 (5.0%)	3 (0.8%)	106 (29.5%)
Until hospital discharge	70 (19.5%)	26 (7.2%)	43 (12.0%)	87 (24.2%)	24 (6.7%)^xx^	22 (6.1%)	12 (3.3%)	112 (31.2%)
Infection (*N* = 82, 22.8%)								
At ED admission	6 (7.3%)	4 (4.9%)	3 (3.7%)	11 (13.4%)	1 (1.2%)	–	–	12 (14.6%)
24 h after ED admission	7 (8.5%)	4 (4.9%)	4 (4.9%)	11 (13.4%)	2 (2.4%)	2 (2.4%)	0 (0.0%)	15 (18.3%)
48 h after ED admission	7 (8.5%)	4 (4.9%)	5 (6.1%)	12 (14.6%)	2 (2.4%)	2 (2.4%)	0 (0.0%)	15 (18.3%)
72 h after ED admission	7 (8.5%)	4 (4.9%)	5 (6.1%)	12 (14.6%)	2 (2.4%)	2 (2.4%)	0 (0.0%)	15 (18.3%)
Until hospital discharge	10 (12.2%)	6 (7.3%)	6 (7.3%)	14 (17.1%)	4 (4.9%)	3 (6.7%)	1 (1.2%)	18 (22.0%)
Sepsis (*N* = 277, 77.2%)								
At ED admission	39 (14.1%)	17 (6.1%)	11 (4.0%)	61 (22.0%)	3 (1.1%)	–	–	64 (23.1%)
24 h after ED admission	44 (15.9%)	18 (6.5%)	25 (9.0%)	71 (25.6%)	8 (2.9%)	14 (5.1%)	1 (0.4%)	83 (30.0%)
48 h after ED admission	50 (18.1%)	19 (6.9%)	28 (10.1%)	71 (25.6%)	13 (4.7%)	16 (5.8%)	1 (0.4%)	87 (31.4%)
72 h after ED admission	53 (19.1%)	19 (6.9%)	30 (10.8%)	75 (27.1%)	13 (4.7%)^x^	16 (5.8%)	3 (1.1%)	91 (32.9%)
Until hospital discharge	60 (21.7%)	20 (7.2%)	37 (13.4%)	73 (26.4%)	20 (7.2%) ^xx^	19 (6.9%)	11 (4.0%)	94 (33.9%)

ED: emergency department; ^x^ of which one patient with all three organ systems failing; ^xx^ of which four patient with all three organ systems failing

In the patients who presented with infection, 14.6% had signs of organ failure at ED admission (Table 3). An additional 3.7% of the patients with infection deteriorated in the first 24 h after admission. Two patients (2.4%) required ICU admission and one patient (1.2%) developed multiple organ failure. In the remainder of the first 72 h, no additional patients deteriorated.

Of the patients with sepsis, 23.1% had signs of organ failure at ED admission (Table 3). Most of them had AKI (14.1%). In the first 24 h after admission, an additional 6.9% of the patients with sepsis deteriorated, mostly due to respiratory failure (+ 5%). An additional 1.8% of the patients deteriorated to multiple organ failure and after 48 h another 1.8% of the patients had developed multiple organ failure. After 72 h, one patient had multiple organ failure in all three organ systems. During the rest of the hospitalization, only 1% of the patients deteriorated additionally. In the remainder of this article we use the first 72 h of admission as timeframe for patient deterioration.

### Age and diabetes associated with higher risk of deterioration

The logistic regression base model for patient deterioration including age, gender and comorbidities yielded an AUC of 0.679 (Table 4). A higher age (odds ratio (OR): 1.02 / year) and a history of diabetes (OR: 1.90) were associated with a higher risk of patient deterioration. Gender and comorbidities other than diabetes were not independent predictors of deterioration.

**Table 4:** Logistic regression models for deterioration within 72 h from admission based on repeated vital sign measurements with a 30-min interval during the first 3 h of ED admission

		Sig.	Odds Ratio (95% CI)	Model statistics		
				Cox & Snell R^2^	AUC (95% CI)	N ^a^
Base model for deterioration within 72 h from admission				.080	.679 (.619; .739)	359 (100%)
Age		.012*	1.022 (1.005; 1.039)			
Gender (0 = male, 1 = female)		.502	0.839 (0.502; 1.402)			
Cardiac disease		.158	1.544 (0.845; 2.820)			
COPD		.159	1.906 (0.777; 4.676)			
Diabetes		.035*	1.902 (1.048; 3.454)			
Chronic kidney disease		.308	1.475 (0.699; 3.111)			
Chronic liver disease		.345	1.493 (0.650; 3.429)			
Organ transplant		.245	1.408 (0.791; 2.509)			
Malignancy		.450	0.807 (0.463; 1.407)			
Base model with heart rate						
HR-M1.	Heart rate at admission	.042*	1.013 (1.000; 1.025)	.091	.683 (.623; .742)	359 (100%)
HR-M2.	Heart rate at admission	.035*	1.015 (1.001; 1.030)	.091	.684 (.624; .743)	358 (99.7%)
	Heart rate change	.463	1.039 (0.938; 1.151)			
HR-M3.	Heart rate at admission	.062	1.013 (0.999; 1.027)	.091	.683 (.624; .743)	359 (100%)
	Heart rate variability	.884	0.998 (0.977; 1.021)			
Base model with mean arterial pressure						
MAP-M1.	MAP at admission	<.001*	0.955 (0.937; 0.972)	.156	.746 (.688; .804)	357 (99.4%)
MAP-M2.	MAP at admission	<.001*	0.940 (0.920; 0.961)	.176	.758 (.701; .815)	355 (98.9%)
	MAP change	.003*	0.873 (0.798; 0.954)			
MAP-M3.	MAP at admission	<.001*	0.941 (0.922; 0.960)	.223	.800 (.750; .850)	357 (99.4%)
	MAP variability	<.001*	1.060 (1.037; 1.084)			
Base model with respiratory rate						
RR-M1.	Respiratory rate at admission	.042*	1.048 (1.002; 1.097)	.075	.663 (.592; .735) ^b^	267 (74.4%)
RR-M2.	Respiratory rate at admission	.004*	1.086 (1.027; 1.148)	.096	.686 (.617; .755) ^b^	242 (67.4%)
	Respiratory rate change	.018*	1.441 (1.063; 1.952)			
RR-M3.	Respiratory rate at admission	.144	1.022 (0.988; 1.071)	.087	.676 (.605; .746) ^b^	267 (74.4%)
	Respiratory rate variability	.063	1.067 (0.996; 1.142)			
Base model with body temperature						
BT-M1.	Body temperature at admission	.607	1.059 (0.845; 1.319)	.083	.680 (.619; .741)	355 (98.9%)
BT-M2.	Body temperature at admission	.880	1.020 (0.786; 1.324)	.080	.681 (.619; .743)	342 (95.3%)
	Body temperature change	.677	0.720 (0.153; 3.385)			
BT-M3.	Body temperature at admission	.962	0.994 (0.790; 1.252)	.090	.683 (.622; .745)	355 (98.9%)
	Body temperature variability	.097	1.389 (0.942; 2.049)			

AUC: area under the receiver operating curve; CI: confidence interval; COPD: Chronic Obstructive Pulmonary Disease; HR: Heart rate, MAP: mean arterial pressure; RR: respiratory rate; BT: body temperature; Sig.: statistical significance; * significant result (*p* < 0.05)

^a^Missing or observations that were constant within the measured time period are excluded from the regression model; ^b^ the AUC of the base model only including patients with respiratory rate at admission was .638

### Vital signs at ED admission are associated with deterioration

**Fig. 2:**
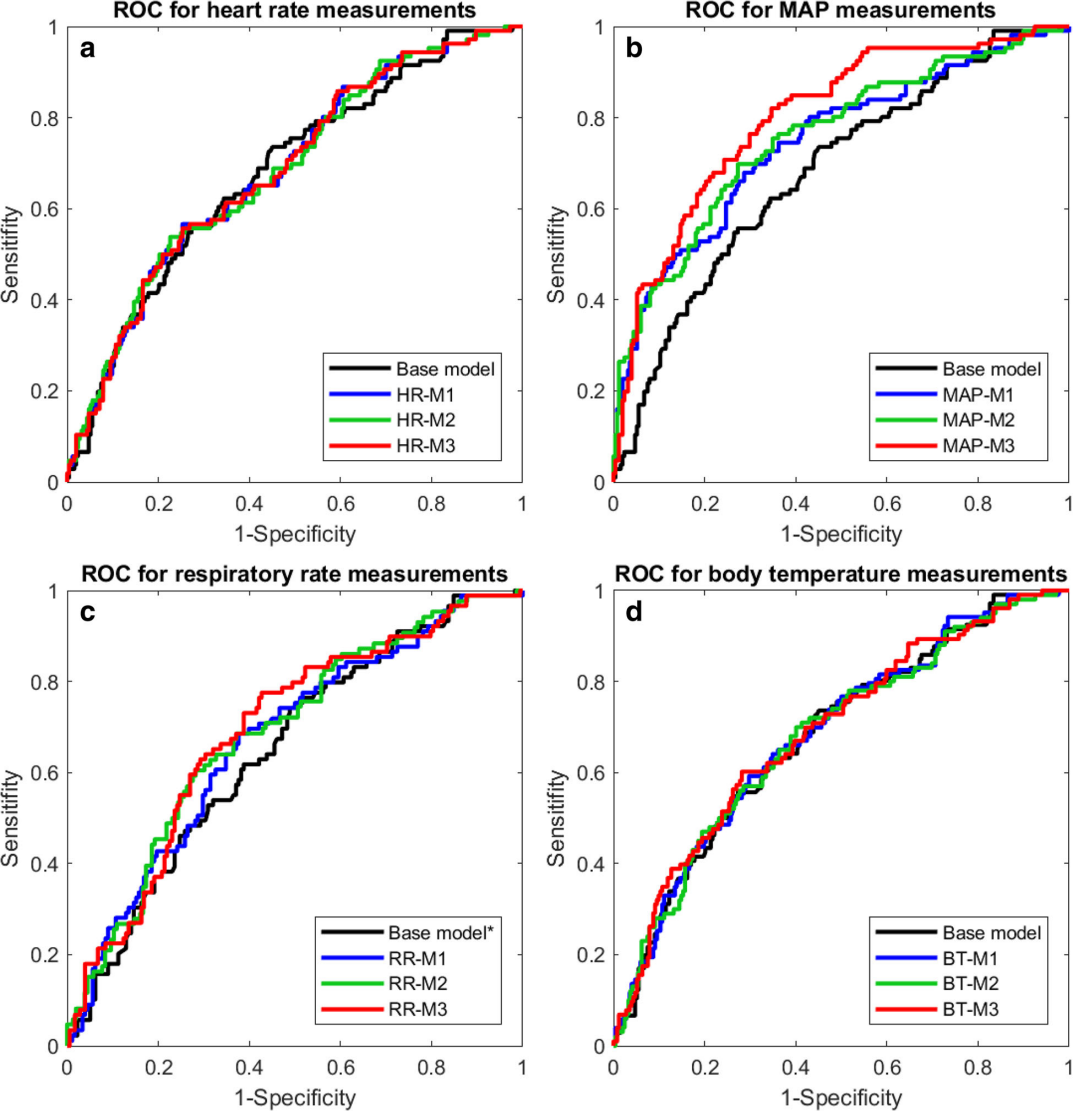
Receiver operating curves of the logistic regression models for patient deterioration using various repeated vital sign measurements in 30-min intervals during the first three hours of the patient’s stay in the emergency department. The base model includes age, gender and comorbidities. Model M1 contains the base model combined with the value of the vital sign at admission, model M2 contains model M1 combined with the change of the vital sign over time, model M3 contains model M1 combined with the variability of the vital. A) the ROC curve for the base model combined with heart rate (HR). B) the ROC curve for the base model combined with mean arterial pressure (MAP). C) the ROC curve for the base model combined with respiratory rate (RR). ^*^ Base model only including patients with respiratory rate at admission (AUC .638). D) the ROC curve for the base model combined with body temperature (BT)

Patients who deteriorated had a lower MAP (*p* < 0.001) and a higher respiratory frequency (*p* = 0.03) at ED admission (Table 1). The base model extended with the patient’s vital signs at ED admission, showed that both a higher heart rate (OR: 1.01/beat per minute; model HR-M1; AUC .683) and a higher respiratory rate (OR: 1.05/respiration per minute; model RR-M1; AUC .663) were associated with a higher risk of deterioration (Table 4, Fig. 2). A higher MAP at ED admission was associated with a lower risk of deterioration (OR: 0.96/mmHg; model MAP-M1; AUC .746). The body temperature at ED admission was not independently associated with deterioration (model BT-M1; AUC .680).

### Repeated vital sign measurements improve the prediction of deterioration

Next to the vital signs at ED admission, the change and variability of the repeated vital signs measurements in the first 3 h in the ED were entered into the base model together with the vital signs at ED admission (Table 4, Fig. 2). An increase in MAP over time was associated with a lower risk of deterioration (OR: 0.873/unit increase; model MAP-M2; AUC .758). An increase in respiratory rate over time was associated with a higher risk of deterioration (OR: 1.441/unit increase; model RR-M2; AUC .686). The changes in heart rate and temperature were not independently associated with deterioration.

Next to the vital signs at ED admission and change over time, a higher variability in MAP (i.e. a higher range) was significantly associated with a higher risk of deterioration (OR: 1.06/mmHg; model MAP-M3; AUC .800; Table 4, Fig. 2). The variability of the other vital signs was not associated with the risk of deterioration.

## Discussion

The aim of our study was to determine whether repeated vital sign measurements in the ED can identify patients with sepsis or infection that will deteriorate within 72 h. We found an increase in MAP over time was associated with a lower risk of deterioration, and a higher variability of the MAP or increase in respiratory rate over time, in combination with their respective values at ED admission, were associated with patient deterioration. Inclusion of repeated MAP measurements resulted in the largest AUC (.800), whereas repeated respiratory rate measurements only slightly improved the predictive capabilities of the logistic regression model over the base model. Repeated measurements of heart rate and body temperature were not associated with patient deterioration.

Our results indicate that changes and variability of the MAP are associated with patient deterioration in ED patients with infection or sepsis. This suggests that keeping a close eye on the MAP during the patients stay in the ED is important. Our study shows that this not only applies to patients with septic shock (only 1.9% of our population), as recommended by the surviving sepsis campaign (SSC) guidelines, but for all patients with sepsis or infection [4].

Apart from our earlier pilot study, little is known about repeated vital sign measurements in patients with infection or sepsis during their stay in the ED in relation to clinical outcomes, patient deterioration and (early) signs of organ failure. Our pilot study showed that vital signs changed significantly during the patient’s stay in the ED, but did not analyse patient deterioration [7]. Henriksen et al.*..* retrospectively found a deterioration of vital signs from the normal to abnormal range within 4–13 h after arrival in 31% of patients in the general ED population, leading to a four times higher 30-day mortality risk [13]. The available studies on vital signs in the ED mostly use only single measurements, mainly at triage [9, 12]. Furthermore, these were often retrospective studies in contrast to our study. Finally, they often included the general ED population and thus a more heterogeneous population. The endpoints and cut-off values differ from study to study, most studies used mortality endpoints, several studies had ICU admission as an endpoint and only a few studies included organ failure [8, 11, 13, 19–22]. The single measurements, heterogeneous patient populations and different endpoints make a direct comparison of those results with our study’s results impossible. Coslovsky et al aimed to develop a prediction model for in-hospital mortality using a model with age, prolonged capillary refill, blood pressure, mechanical ventilation, oxygen saturation index, GSC and the APACHEII diagnostic category in a cohort that contained 15% patients with infection among which 7.3% with sepsis. Their model had an AUC of 0.92, although, it should be noted that their model was based on a heterogeneous patient population, single measurements and a combination of multiple vital signs [23]. Yamamoto et al. found an association between low body temperature (< 36 °C) at ED admission and higher 30 day in-hospital mortality risk in patients with suspected sepsis [24]. In our study, we did not find an association between body temperature and deterioration. Furthermore, it should be noted that the in-hospital mortality in our study (3.3%) is much lower than in the study of Yamamoto (9.6%). In summary, available studies did not specifically investigate ED patients with infection or sepsis, mostly used single vital sign measurements (at triage) and primarily had mortality or ICU admission endpoints.

Early warning scores (EWS), like the national early warning score (NEWS) and many variants and related scores, are increasingly being used throughout healthcare. These EWS commonly contain a combination of various vital sign parameters, supplemented with laboratory values or other items, where each item is scored at certain thresholds. Early warning scores are mostly used as ‘track-and-trigger’ systems to trigger the nurse to call the physician or a rapid response team, or to predict a high risk of mortality or ICU admission [25, 26]. The many different EWS and patient populations, in which they have been validated, make it difficult to compare their performance. However, a recent review by Nannan Panday et al. showed that the NEWS score was the best to predict mortality or ICU admission in the general ED population and the modified early warning score was the best in patients with suspected infection or sepsis [25]. Their performance (AUC) was in the same range as we found for our repeated blood pressure measurements (MAP). However, it should be noted that we used only a single vital sign repeated measurement and had a composite outcome of patient deterioration, which included signs of organ dysfunction. Another recent study by Kivipuro et al. showed that the NEWS score was significantly higher before ICU admission when a patient was transferred from the ward to the ICU, compared to the NEWS score of the same patient at the ED [27]. In our hospital, modified early warning scores (MEWS) are taken at admission to the ward and thereafter three times per day. Deterioration of the MEWS score triggers an early response team. Further research is needed to clarify whether repeated vital sign measurements in combination with repeated early warning scores are useful in the detection of patient deterioration in patients with sepsis or infection.

We have shown that almost 30% of the patients presenting to the ED with suspected infection or sepsis deteriorated within 72 h of admission and over 28% of the patients showed signs of (multiple) organ failure despite treatment. Our results show that 18.3% of the patients with infection, 32.9% of the patients with sepsis and in total 29.5% of the patients deteriorated within 72 h (Table 4). Glickman et al. showed that almost 23% of patients with uncomplicated sepsis progress to severe sepsis or septic shock within 72 h from admission [1]. Although a direct comparison cannot be made because of a different population and different endpoints, these results clearly show that a large part of the patients with infection deteriorate in the first days in the hospital and develop (severe) sepsis. Therefore, we question whether the introduction of the recent Sepsis-3 definitions, in which infection or uncomplicated sepsis are no longer part of the sepsis severity spectrum, will lead to better patient care [28]. We would like to emphasise that it is important to properly monitor and treat all patients with infection or sepsis in the ED. Since sepsis-related mortality has dramatically reduced over the past two decades, we believe that early detection or prevention of organ failure is where the future focus of infection/sepsis research should be, since there is a lot to gain [29].

The 30-min measurement interval in the current study was arbitrarily chosen, since there is no standard on how often vital signs should be measured in the ED and only little research has been conducted on this topic. Descriptive studies in the general ED population have shown that the time between two measurements is between 67 and 130 min and that a higher illness severity results in more frequent measurements [10, 30]. We believe that these measurement intervals are not representative for patients with infection or sepsis, however, there are no specific guidelines on how often vital signs should be measured in these patients [13]. The 30-min measurement interval in our study was much more frequent than the median intervals reported by Johnson and Lambe [10, 30]. A higher measurement frequency might provide even more information about deterioration, although this might lead to a higher burden on the patient and staff. Therefore, we recommend continuous measurement of vital signs on a beat-to-beat level, preferably automated with the use of bed-side patients monitors or wearable devices [3]. Our next step, as a follow-up of this study, is to shorten the measurement interval to a beat-to-beat interval with heart rate variability (HRV) measured using bed-side patient monitors in the SepsiVit study [3]. As we have shown, a substantial number of patients deteriorate in the first days from admission. In the currently running SepsiVit study, we will extend the measurements beyond the boundaries of the ED towards the nursing wards during the first 48 h of hospitalization. During this period, we will investigate whether the combination of HRV with monitoring on the nursing wards can provide an early warning of patient deterioration. Such an early warning could provide a possible opportunity for intervention in the future.

### Strengths and limitations

To the best of our knowledge this is the first study that prospectively investigated the relation between repeated vital sign measurements and patient deterioration in the ED in patients with infection or sepsis. We did not only use the common mortality and ICU admission endpoints, but also included signs of organ failure in our composite patient deterioration endpoint. Vital signs can be easily measured with equipment readily available in every ED. The repeated vital sign measurements in our study were obtained specifically by a trained member of our research staff, which minimized the amount of missing data. However, in spite of the prospective study design, 92 (25%) respiratory rate measurements were not recorded at triage by the triage nurse. It is well-known that respiratory rate is the most frequently missing vital sign, unfortunately our study is no exception [31]. These missing respiratory rate measurements at triage limit the power of our logistic regression models that include respiratory rate (RR-M*x*; Table 4).

Another limitation of our study is that it is a single centre study in an academic tertiary care teaching hospital. This may limit the generalizability to other patient populations, especially since our population contains a high number of patients with a history of organ transplantation (Table 2). However, a history of organ transplantation was not independently associated with patient deterioration in our models (Table 4). Therefore, we believe that the specific patient population did not have a substantial influence on our results. We did not design the study to analyse combinations of multiple vital signs in our models, since we were interested in identifying which repeated vital sign measurements are helpful in predicting patient deterioration and not in the best combination of vital signs. We acknowledge that a combination of repeated vital signs may provide even more information in future studies, perhaps in combination with repeated early warning scores.

### Clinical implications

We have shown that more than one in four patients presenting to the ED with suspected infection or sepsis deteriorated within 72 h of admission and showed signs of organ failure. These were not exclusively patients with sepsis at admission, but one in five patients that presented to the ED with infection only. Although the organ failure generally did not result in mortality, organ failure may even be preventable or treatable. Our results show that repeated vital sign measurements (especially blood pressure) at the ED is a predictor of patient deterioration and might result in a reduction of organ failure related morbidity. It is thus important to reassess patient at the ED frequently, including measurement of vital signs, as is done on the wards with early warning scores [29]. Although it is known that patient deterioration is often preceded by changes in vital signs several hours before the event, these signs are frequently missed on general wards [25, 27, 32]. At this moment, we are conducting a subsequent study (SepsiVit study) with 48 h of continuous vital sign measurements at the ED and on the general wards to test the hypothesis that repeated vital sign measurements at the general ward (with high frequency) is better in the prediction of patient deterioration than the currently used systems [3]. Until this information from the SepsiVit study becomes available, we assess patients at the ED frequently, including repeated vital sign measurements.

## Conclusions

Repeated measurement of vital signs in the ED are better at identifying patients at risk for deterioration within 72 h from admission than single vital sign measurements at ED admission. Repeated measurements of MAP and respiratory rate are associated with patient deterioration. Since almost one third of patients presenting with infection or sepsis to the ED deteriorate within 72 h, repeated vital sign measurements may be an important way to guarantee early identification of deterioration.

## Abbreviations

AKI: Acute kidney injury
AUC: Area under the receiver operator curve
BT: Body temperature
COPD: Chronic obstructive pulmonary disease
ED: Emergency department
GCS: Glasgow coma score
HR: Heart rate
HRV: Heart rate variability
ICU: Intensive care unit
KDIGO: Kidney disease improving global outcomes
MAP: Mean arterial pressure
METc: Institutional review board
OR: Odds ratio
RR: Respiratory rate
SBP: Systolic blood pressure
SCPD: Sepsis clinical pathway database
SpO_2_: Peripheral oxygen saturation
SSC: Surviving sepsis campaign
USA: United States of America
